# SENP3 Promotes an Mff-Primed Bcl-x_*L*_-Drp1 Interaction Involved in Cell Death Following Ischemia

**DOI:** 10.3389/fcell.2021.752260

**Published:** 2021-10-15

**Authors:** Chun Guo, Keri L. Hildick, Juwei Jiang, Alice Zhao, Wenbin Guo, Jeremy M. Henley, Kevin A. Wilkinson

**Affiliations:** ^1^School of Biosciences, University of Sheffield, Sheffield, United Kingdom; ^2^School of Biochemistry, University of Bristol, Bristol, United Kingdom; ^3^School of Medical Sciences, University of Manchester, Manchester, United Kingdom; ^4^Faculty of Science, Centre for Neuroscience and Regenerative Medicine, University of Technology Sydney, Ultimo, NSW, Australia

**Keywords:** Drp1, Bcl-x_*L*_, SENP3, Mff, SUMOylation, ischemia

## Abstract

Dysregulation of the mitochondrial fission machinery has been linked to cell death following ischemia. Fission is largely dependent on recruitment of Dynamin-related protein 1 (Drp1) to the receptor Mitochondrial fission factor (Mff) located on the mitochondrial outer membrane (MOM). Drp1 is a target for SUMOylation and its deSUMOylation, mediated by the SUMO protease SENP3, enhances the Drp1-Mff interaction to promote cell death in an oxygen/glucose deprivation (OGD) model of ischemia. Another interacting partner for Drp1 is the Bcl-2 family member Bcl-x_*L*_, an important protein in cell death and survival pathways. Here we demonstrate that preventing Drp1 SUMOylation by mutating its SUMO target lysines enhances the Drp1-Bcl-x_*L*_ interaction *in vivo* and *in vitro*. Moreover, SENP3-mediated deSUMOylation of Drp1 promotes the Drp1-Bcl-x_*L*_ interaction. Our data suggest that Mff primes Drp1 binding to Bcl-x_*L*_ at the mitochondria and that Mff and Bcl-x_*L*_ can interact directly, independent of Drp1, through their transmembrane domains. Importantly, SENP3 loss in cells subjected to OGD correlates with reduced Drp1-Bcl-x_*L*_ interaction, whilst recovery of SENP3 levels in cells subjected to reoxygenation following OGD correlates with increased Drp1-Bcl-x_*L*_ interaction. Expressing a Bcl-x_*L*_ mutant with defective Drp1 binding reduces OGD plus reoxygenation-evoked cell death. Taken together, our results indicate that SENP3-mediated deSUMOlyation promotes an Mff-primed Drp1-Bcl-x_*L*_ interaction that contributes to cell death following ischemia.

## Introduction

Ischemia, the restriction in blood supply to tissues, is a leading cause of death and disability. Standard clinical treatment is to restore blood supply as soon as possible to limit infarct size and reduce mortality. Paradoxically, however, this can cause further damage, referred to as reperfusion injury (Hausenloy and Scorrano, 2007). The pathological consequences of ischemia/reperfusion (I/R) injury are complex, but severely impaired mitochondrial integrity invariably precedes cell death. A well-characterized pathway involves reactive-oxygen species and Ca^2+^-mediated activation of the mitochondrial inner membrane permeability transition pore, which leads to cell necrosis (Halestrap, 2010; Tait and Green, 2010; Landes and Martinou, 2011; Pasdois et al., 2012). Additionally, it is becoming increasingly clear that mitochondrial outer membrane (MOM) proteins that regulate fission induce cell death via either modulating mPTP opening or through apoptotic mechanisms (Halestrap, 2009, 2010; Tait and Green, 2010; Landes and Martinou, 2011; Pasdois et al., 2012; Whelan et al., 2012). Specifically, this involves mitochondrial fission regulated by the cytosolic dynamin family large GTPase Drp1, which can be recruited to the MOM by interacting with the docking proteins Mff, MiD49, MiD51 and possibly Fis1 (Atkins et al., 2016). The recruited Drp1 molecules polymerise in a spiral around the mitochondria and cause scission of the mitochondria by GTP hydrolysis (Elgass et al., 2013). Inhibition of Drp1 using the GTPase blocker dynasore (Macia et al., 2006) or the Drp1-selective inhibitor Mdivi-1 (Cassidy-Stone et al., 2008) can protect cells against I/R injury *in vitro* and *in vivo* (Grohm et al., 2012; Sharp et al., 2015; Wang et al., 2018), and reduced Drp1 localization at mitochondria is cytoprotective (Ong et al., 2010; Grohm et al., 2012; Din et al., 2013; Sharp et al., 2014). Thus, active Drp1 localized at mitochondria plays a key role in mediating cell death following extreme stress, but the upstream triggers and downstream effector systems that underpin these effects are largely unknown.

Intriguingly, animals that hibernate endure prolonged ischemia and subsequent reperfusion but emerge from hibernation torpor undamaged (Andrews, 2007). This natural phenomenon appears at least partially attributable to the cytoprotective effects of increased SUMOylation during torpor (Lee et al., 2007, 2012). SUMOylation is the covalent conjugation of a Small Ubiquitin-like MOdifier protein (SUMO) to lysine residue(s) in target proteins. Currently three conjugatable SUMO paralogues (SUMO-1-3) have been identified in humans. SUMO-2 and SUMO-3 are very similar, differing in only three amino acid residues, and are therefore collectively termed SUMO-2/3, but SUMO-1 differs from SUMO-2/3. It is generally accepted that SUMO-1 is important for normal cell function and maintenance, whereas, in contrast, SUMO-2/3 appears to be essential in cell stress pathways (Guo and Henley, 2014). In agreement with observations made in hibernating animals, increased SUMO-2/3-lyation is readily detectable in cerebral ischemia models (Cimarosti et al., 2008; Yang et al., 2008a, b; Loftus et al., 2009), and microRNA-mediated knockdown of SUMO-2/3 substantially reduces cell survival following ischemic stress (Datwyler et al., 2011), highlighting the critical role for SUMO-2/3 in preventing cell death.

Protein SUMOylation is reversible, and target proteins can be deSUMOylated by a number of identified SUMO protease(s) (Hickey et al., 2012). The largest and best characterized family of SUMO proteases is that of the sentrin-specific proteases (SENPs), composed of six members (SENP1–3 and 5–7) with each having distinct deconjugation preference toward specific SUMO paralog(s) (Hickey et al., 2012). Both SENP1 and SENP2 have deconjugation activity for SUMO-1 and SUMO-2/3, whereas SENP3 and SENP5 have specific activity for SUMO-2/3 deconjugation (Gong and Yeh, 2006). However, specific targets and pathophysiological roles for SENPs in cell stress responses are largely unknown.

In previous work we uncovered a cytoprotective pathway in which degradation of SENP3 upon ischemia (modeled by oxygen/glucose depravation, OGD) protects the SUMO-2/3-ylation status of the GTPase Drp1, a well-established SUMO target protein (Figueroa-Romero et al., 2009), reducing Drp1 localization at mitochondria to enhance cell survival (Guo et al., 2013). However, during reoxygenation following OGD, SENP3 levels recover, reducing Drp1 SUMOylation and enhancing Drp1 localization at mitochondria to promote cell death (Guo et al., 2013).

Our further work revealed that SENP3-mediated deSUMOylation selectively enhances Drp1 interaction with the docking protein Mff, thereby promoting Drp1 mitochondrial localization and eventually contributing to cell death evoked by reoxygenation following OGD (Guo et al., 2017). These results highlight that SENP-mediated deSUMOylation is a regulator of protein-protein interactions that can significant impact on cellular processes, including mitochondrial dynamics and cell death.

Interestingly, in addition to its association with Mff, Drp1 also binds the Bcl-2 family member Bcl-x_*L*_ (Jonas, 2014). The Drp1-Bcl-x_*L*_ interaction appears to be important for synaptogenesis (Li et al., 2008) and modulation of neurotransmitter vesicle endocytosis in cultured primary rat hippocampal neurons (Li et al., 2013). However, it is unknown whether SENP3-mediated deSUMOylation regulates the Drp1-Bcl-x_*L*_ interaction, and whether this interaction has a role in cell death and survival pathways (Michels et al., 2013).

Here, using deletion mutagenesis and amino acid substitution approaches in our established ischemia model, we systematically explored the impact of changes in Drp1 SUMOylation on the Drp1-Bcl-x_*L*_ interaction, and the roles of the deSUMOylating enzyme SENP3 and Mff in regulating the Drp1-Bcl-x_*L*_ interaction. We examined the dynamic changes in the Drp1-Bcl-x_*L*_ interaction following ischemia and assessed the functional consequence of the loss of the Drp1-Bcl-x_*L*_ interaction in cell death evoked by reoxygenation following OGD.

## Materials and Methods

### Plasmids and Mutagenesis

DNA constructs encoding Flag-SENP3, GST-Mff, GST-Mff ΔN50, YFP-Drp1, YFP-Drp1^*R*^, YFP-Drp1 4KR, YFP-Drp1^*R*^ 4KR, and YFP-Drp1^*R*^Δ15 have been described previously (Guo et al., 2013, 2017). Drp1-His was provided by M. Matsushita (Han et al., 2008). Drp1 Δ15-His was made by PCR-based mutagenesis. Flag-Mff was generated by insertion of the relevant cDNA into the BamH1/Not1 sites of pcDNA3-Flag. GST-Mff ΔC deletion mutant (residues 1–322) was generated by insertion of the relevant cDNA into the BamH1/Not1 sites of pEBG. GST-Mff ΔRR (residues 1–340) was generated by PCR-based mutagenesis to remove the DNA sequence encoding the final two residues (R341 and R342) at the C-terminus of Mff. RFP-Mff TM was generated by PCR-based mutagenesis to fuse the cDNA sequence encoding the Mff TM (residues 323–340) to the C-terminus of RFP immediately before the stop codon (TAA) in pcDNA3-RFP (Addgene Plasmid #13032; provided by D. Golenbock). Mammalian expression DNA constructs encoding HA-Bcl-x_*L*_ and *E. coli* expression construct (pTXB1) encoding Bcl-x_*L*_, were provided by D. R. Green (Llambi et al., 2011). Bcl-x_*L*_-YFP was generated by PCR-based insertion of the relevant cDNA into the EcoRI/NotI sites of pcDNA3-YFP (Addgene Plasmid #13033; provided by D. Golenbock) (Guo et al., 2017). Bcl-x_*L*_ ΔC deletion mutant-YFP (residues 1–211), Bcl-x_*L*_ ΔC7 deletion mutant-YFP (residues 1–226), and Bcl-x_*L*_ ΔN deletion mutant-YFP (lacking residues 2–76) were generated by insertion of the relevant cDNAs into the EcoRI/NotI sites of pcDNA3-YFP, respectively. YFP-Bcl-x_*L*_ TM was generated by PCR-based mutagenesis to fuse the cDNA sequence encoding Bcl-x_*L*_ TM (residues 210–226) to the C-terminus of YFP immediately before the stop codon (TAA) in pcDNA3-YFP. Bcl-x_*L*_ M2 (mutant)-YFP was generated by PCR-based mutagenesis to mutate the DNA sequence encoding three residues (W188S, D189V, and F191C) in the BH2 domain of Bcl-x_*L*_ using a pair of primers as previously described (Li et al., 2013).

### Protein Expression and Purification and Histidine Pulldown-Based Binding Assay

Bcl-x_*L*_ was first purified from the vector pTXB1 using *E. coli* BL21 Star (DE3) cells as a fusion protein with intein/chitin-binding domain using a chitin resin, and Bcl-x_*L*_ was then cleaved from the resin in a buffer containing 20 mM Tris pH 8.0, 0.5 M NaCl, 1 mM EDTA, protease inhibitors (Roche), and 50 mM DTT for 48 h using IMPACT^TM^ Kit (New England Biolabs) as described previously (Llambi et al., 2011). Drp1 WT-His or Drp1 Δ15-His mutant were produced from the vector pET21a using *E coli* BL21 Star (DE3) cells (Invitrogen) as described previously (Guo et al., 2017) and purified on Ni^2+^-NTA beads (Qiagen). To detect the physical interaction between Bcl-x_*L*_ and Drp1, an *in vitro* binding assay was performed. Briefly, equal amount of Bcl-x_*L*_ were incubated with Ni^2+^-NTA beads or Ni^2+^-NTA beads bound with His-tagged Drp1 WT or Δ15 mutant in a binding buffer containing 10 mM HEPES, 1 mM EDTA (pH 7.4), 150 mM NaCl, on a rotating platform for 1 h at 4°C. At the end of the incubation, the beads were spun down, washed three times with a buffer containing 10 mM HEPES, 1 mM EDTA (pH 7.4), 150 mM NaCl plus 0.2% CHAPS, and resuspended in SDS sample buffer for immunoblot analysis.

### Cell Culture

HEK293 cells were grown in Dulbecco’s modified Eagle’s medium (Lonza) containing 10% fetal bovine serum, 5 mM glutamine, and 100 units/ml penicillin/streptomycin at 37°C in humidified ambient air supplemented with 5% CO_2_ as described previously (Guo et al., 2013, 2017).

### DNA and siRNA Transfections

DNA, siRNA, or DNA & siRNA were transfected into HEK293 cells using jetPRIME (Polyplus Transfection). siRNA duplexes used were as follows: human SENP3 siRNA (Santa Cruz, sc-44451) and human Bcl-xL-specific siRNA (Eurofins MWG Operon; target sequence GCGUAGACAAGGAGAUGC).

### Subcellular Fractionation

Mitochondrial and cytosolic fractions from HEK293 cells were prepared as described previously (Guo et al., 2013, 2017).

### Oxygen-Glucose Deprivation and Reoxygenation

OGD was conducted within a SCI-tive hypoxia workstation (Baker Ruskinn) under the settings of 0.1% O_2_ and 5% CO_2_ at 37°C. Briefly, DMEM without glucose containing 0.1% FBS (Invitrogen) and 5 mM glutamine was deoxygenated for 2 h within the hypoxia workstation. HEK293 cells were then washed with the deoxygenated DMEM, and the cells were replaced with the medium and maintained in the hypoxia workstation for 2 h. In parallel, normal culture medium was removed from control cells, the cells were washed with fresh DMEM containing glucose, 0.1% FBS, and 5 mM glutamine, and replaced with the medium and maintained in a normoxic cell culture incubator. For reoxygenation experiments HEK293 cells were moved from the hypoxia workstation and kept at 37°C in humidified ambient air supplemented with 5% CO_2_ for a further 24 h.

### Lactate Dehydrogenase (LDH) Assay

Lactate dehydrogenase (LDH) levels in conditioned culture media were examined using a Lactic Dehydrogenase Based *In Vitro* Toxicology Assay Kit (Sigma) as described previously (Guo et al., 2013, 2017). Values presented in each histogram are representative of at least three independent experiments conducted using different cell populations.

### Preparation of Whole Cell Lysates, Y/RFP-Trap, SUMO-2/3-Trap, GST-Pulldown, and Immunoprecipitation

Whole cell lysates were prepared by lysing cells with a buffer containing 20 mM Tris, pH 7.4, 137 mM NaCl, 25 mM β-glycerophosphate, 2 mM sodium pyrophosphate, 2 mM EDTA, 1% Triton X-100, 0.1% SDS, 10% glycerol, and 1 × protease inhibitor cocktail (Roche). Whole cell lysates were incubated with GFP-Trap-A beads or RFP-Trap Agarose beads (ChromoTek) to enrich and isolate Y/RFP or Y/RFP-tagged proteins as described previously (Guo et al., 2017). Whole cell lysates were incubated with SUMO-2/3 affinity agarose beads (ASM24; Cytoskeleton) at 4°C for overnight to enrich and isolate SUMO-2/3 or SUMO-2/3 conjugates. Whole cell lysates were incubated with glutathione-sepharose 4B beads (Generon) to enrich and isolate GST or GST-tagged proteins as described previously (Guo et al., 2017). To immunoprecipate endogenous Mff, Bcl-x_*L*_ or Drp1, whole cell lysates were incubated overnight with an Mff rabbit polyclonal antibody (17090-1-AP, Proteintech), a Bcl-x_*L*_ rabbit monoclonal antibody (54H6, Cell Signaling) or a Drp1 rabbit monoclonal antibody (D6C7, Cell Signaling), pre-conjugated to protein-A beads (Sigma), respectively. The protein-A beads were spun down, washed three times and resuspended in SDS sample buffer for immunoblot analysis.

### Immunoblotting

Samples were resolved by SDS-PAGE (10–15% gels), transferred to Immobilon-P membranes (Millipore Inc.) and immunoblotted as indicated. Primary antibodies were used to detect Bcl-x_*L*_ (54H6, 1:1,000 dilution, Cell Signaling; 2H12, 1:500 dilution, Invitrogen), COX IV (3E11, 1:20,000 dilution, Cell Signaling), Drp1 (D6C7, 1:1,000 dilution, Cell Signaling), Flag (66008-3-Ig, 1:1,000 dilution, Proteintech), GAPDH (sc-365062, 1:1,000 dilution, Santa Cruz biotechnology), GFP (sc-8334, 1:1,000 dilution, Santa Cruz biotechnology), HA (51064-2-AP, 1:1,000 dilution, Proteintech), Mff (17090-1-AP, 1:2,000 dilution, Proteintech), PARP (46D11; 1:1,000 dilution, Cell Signaling), RFP (3F5, 1:1,000 dilution, ChromoTek), SENP3 (D20A10, 1:10,000 dilution, Cell Signaling), and β-actin (A2228, 1:20,000 dilution, Sigma). Immune complexes were detected either using HRP-conjugated secondary antibodies (Sigma) or an HRP-conjugated VeriBlot secondary antibody (ab131366, Abcam; for immunoblotting involving IP samples) followed by enhanced chemiluminescence (GE Healthcare Amersham), or using fluorescent secondary antibodies (Thermo Fisher Scientific) by a LI-COR imaging system. Each immunoblot presented is representative of at least three independent experiments carried out using different cell populations.

### Cell Imaging Assay

Cells were fixed for 12 min at room temperature using 4% paraformladehyde/PBS with 5% sucrose. Cell imaging was performed as previously described (Guo et al., 2013).

### Statistics

Graph plotting and statistical analysis were conducted using Graphpad Prism software (Graphpad Inc.). A two-tailed paired Student’s *t*-test was used for comparisons between two data sets, and one-way analysis of variance followed by Tukey’s multiple comparisons test was used for comparison of multiple data sets. All values are expressed as mean ± SEM following the normalization in relation to the control level.

## Results

### The SUMOylation Status of Drp1 Regulates the Drp1-Bcl-x_*L*_ Interaction

**FIGURE 1:**
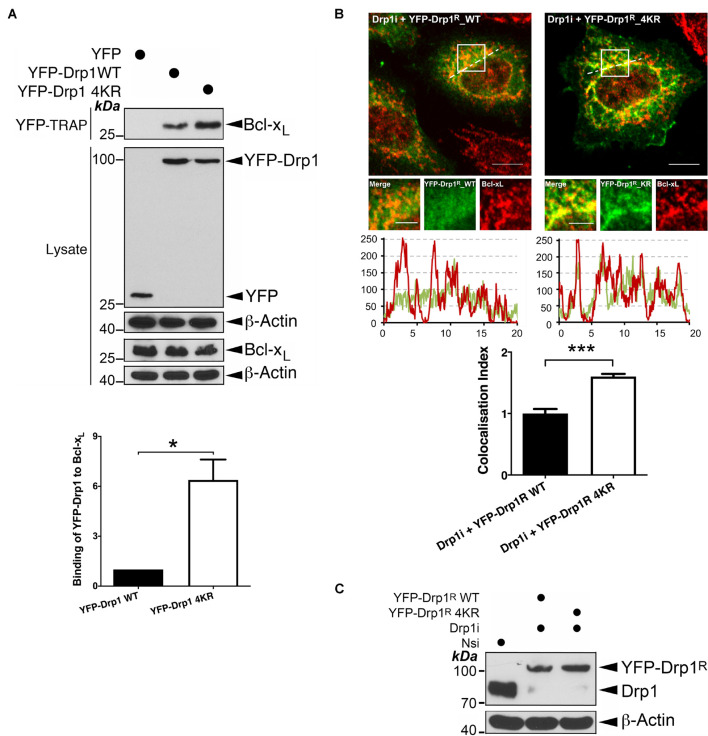
Changes in the SUMOylation status of Drp1 regulate its interaction with Bcl-x_*L*_. **(A)** Ablation of Drp1 SUMOylation results in increased interaction between Drp1 and Bcl-x_*L*_. YFP, YFP-Drp1 WT or YFP-Drp1 4KR mutant were introduced into HEK293 cells. Lysates were subjected to pulldown using YFP-Trap for YFP or YFP-Drp1. YFP-Trap and lysate samples were immunoblotted with Bcl-x_*L*_, GFP, and β-actin antibodies (*n* = 3; *p < 0.05). **(B)** Ablation of SUMOylation in Drp1 results in increased colocalization with endogenous Bcl-x_*L*_. Either YFP-Drp1^*R*^ wild type (WT) or non-SUMOylatable Drp1^*R*^ 4KR mutant were introduced into HeLa cells following knockdown of endogenous Drp1 (*n* = 29 cells for Drp1i+ YFP-Drp1^*R*^ WT and *n* = 26 cells for Drp1i+ YFP-Drp1^*R*^ 4KR; ***p < 0.001). **(C)** Immunoblots (lower panel) confirm knockdown of Drp1 and replacement of YFP-Drp1^*R*^ WT or 4KR mutant in HeLa cells (Nsi, Non-specific siRNA; Drp1i, Drp1 siRNA).

In agreement with previous reports (Li et al., 2013, 2008), we detected an interaction between Bcl-x_*L*_ and Drp1 (Figure 1A and Supplementary Figure 1). Interestingly, compared to wild type (WT), non−SUMOylatable Drp1 4KR mutant shows significantly enhanced association with Bcl-xL (Figure 1A), suggesting that preventing Drp1 SUMOylation promotes its interaction with Bcl-x_*L*_. Consistent with our biochemical results, imaging analysis revealed significantly increased co-localization between RNAi-resistant non−SUMOylatable Drp1 4KR mutant and Bcl-x_*L*_ in Drp1 knockdown HeLa cells, compared to WT Drp1 (Figures 1B,C). Collectively, these results indicate that the Drp1-Bcl-x_*L*_ interaction is regulated by the SUMOylation status of Drp1.

### Bcl-x_*L*_ Binds Directly to Drp1

**FIGURE 2:**
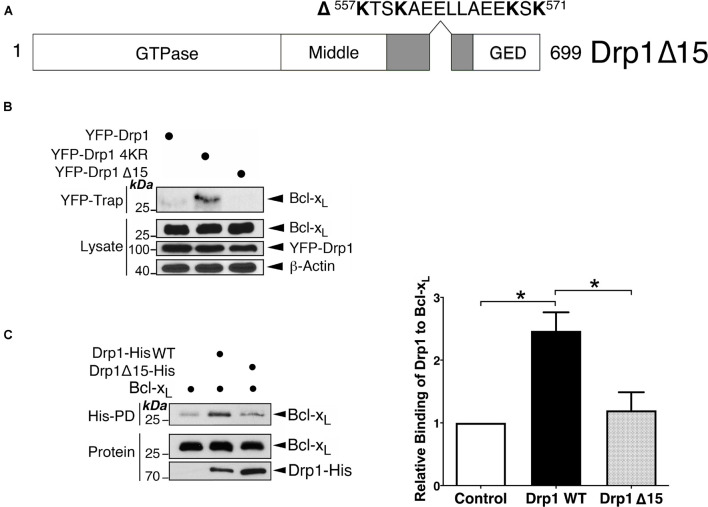
The sequence containing the SUMOylation sites of Drp1 is important for the Bcl-x_*L*_-Drp1 interaction. **(A)** The schematic illustrates a Drp1 mutant in which a 15-residue sequence containing 4 SUMOylatable lysines within the variable region was removed. **(B)** Removal of the SUMOylated region in Drp1 results in decreased interaction between Drp1 and Bcl-x_*L*_ *in vivo*. YFP-Drp1 WT, Drp1 4KR, or Drp1 Δ15 were expressed in HEK293 cells. YFP-Trap and lysate samples were immunoblotted with Bcl-x_*L*_, GFP, and β-actin antibodies. **(C)** Removal of the SUMOylatable region in Drp1 results in decreased interaction between Drp1 and Bcl-x_*L*_ *in vitro*. His-tagged Drp1 or His-tagged Drp1 Δ15 were incubated with Bcl-x_*L*_ *in vitro*. Bcl-x_*L*_ associated with Drp1-His was detected using immunoblotting following His-pulldown (*n* = 3; *p < 0.05).

SUMOylation of Drp1 occurs within a conserved sequence of 15 amino acid residues containing four target lysines required for SUMO conjugation located in the variable region (VR) (Figure 2A; Guo et al., 2017), which is crucial for mitochondrial localization of this large GTPase (Fröhlich et al., 2013). We reasoned that, if this VR sequence is the interface between Drp1 and Bcl-x_*L*_, modification by SUMO might sterically hinder this protein-protein interaction. Moreover, removing this 15 residue sequence altogether (Drp1Δ15 mutant) may prevent Bcl-x_*L*_ binding to Drp1. As expected, endogenous Bcl-x_*L*_ did not associate with the Drp1Δ15 mutant (Figure 2B), indicating the importance of this sequence for Drp1-Bcl-x_*L*_ association and suggesting that fully SUMOylation of Drp1 may completely prevent Drp1 association with Bcl-x_*L*_. Previous studies have not identified whether the Drp1-Bcl-x_*L*_ interaction results from the direct interaction of the two proteins (Li et al., 2008, 2013). We therefore performed *in vitro* binding assays, which demonstrated the physical interaction between Bcl-x_*L*_ and Drp1, and that the Drp1Δ15 mutant no longer interacts with Bcl-x_*L*_ (Figure 2C). Collectively, these results suggest that although direct binding of Bcl-x_*L*_ to Drp1 needs the sequence containing the SUMO conjugation sites in Drp1, SUMO modification of one or more of the lysines within this stretch of amino acid downregulates the Bcl-x_*L*_-Drp1 interaction.

### SENP3 Promotes the Bcl-x_*L*_-Drp1 Interaction

**FIGURE 3:**
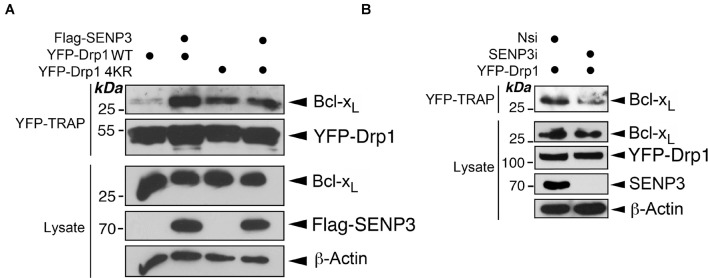
SENP3 promotes the Drp1-Bcl-x_*L*_ interaction. **(A)** Overexpression of SENP3 increases the interaction between Drp1 and Bcl-x_*L*_. Either *pcDNA3* or Flag-SENP3 together with a construct expressing either YFP-Drp1 WT or YFP-Drp1 4KR mutant were transfected into HEK293 cells. YFP-Trap and lysate samples were immunoblotted with Bcl-x_*L*_, GFP, and β-actin antibodies. **(B)** Knockdown of SENP3 decreases the interaction between Drp1 and Bcl-x_*L*_. Either Nsi or SENP3 siRNA together with a construct expressing either YFP-Drp1 or YFP-Drp1 4KR mutant were introduced into HEK293 cells. YFP-Trap and lysate samples were immunoblotted with Bcl-x_*L*_, GFP, and β-actin antibodies.

Drp1 is deSUMOylated by SENP3 (Guo et al., 2013, 2017). Therefore, we investigated if SENP3 regulates the Bcl-x_*L*_-Drp1 interaction. As expected, overexpression of SENP3 enhances Bcl-x_*L*_ interaction with Drp1 WT, but not non-SUMOylatable Drp1 in HEK293 cells (Figure 3A). In contrast, SENP3 knockdown reduces Bcl-x_*L*_ interaction with YFP-Drp1 overexpressed in HEK293 cells (Figure 3B). These data indicate that SENP3-mediated deSUMOylation of Drp1 promotes Drp1-Bcl-x_*L*_ complex formation.

### Mitochondrial Fission Factor Primes the Drp1-Bcl-x_*L*_ Interaction at Mitochondria

**FIGURE 4:**
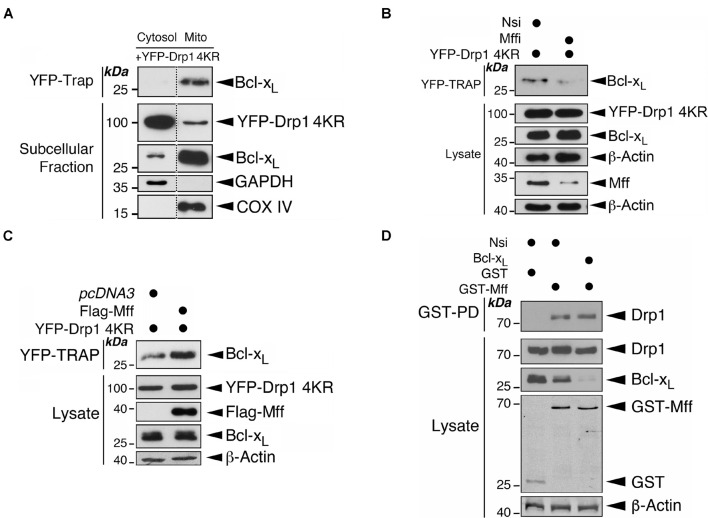
Mff primes the binding of Drp1 to Bcl-x_*L*_ on the mitochondria. **(A)** Drp1 mainly interacts with Bcl-x_*L*_ at mitochondria. YFP-Drp1 4KR mutant was transfected into HEK293 cells. YFP-Drp1 4KR mutant was pulled down from the cytosolic fraction or mitochondrial fraction using YFP-Trap. YFP-Trap and subcellular fraction samples were immunoblotted with Bcl-x_*L*_, GFP, GAPDH and COX IV antibodies. Removal of unrelated lanes are indicated by dotted lines. **(B)** Mff knockdown decreases the interaction between Bcl-x_*L*_ and Drp1. Nsi or Mff siRNA (Mffi) were transfected into HEK293 cells expressing the YFP-Drp1 4KR mutant. YFP-Trap and lysate samples were immunoblotted with Bcl-x_*L*_, GFP, Mff, and β-actin antibodies. **(C)** Mff overexpression increases the interaction between Bcl-x_*L*_ and Drp1. Flag-Mff was transfected into HEK293 cells expressing the YFP-Drp1 4KR mutant. YFP-Trap and lysate samples were immunoblotted with Bcl-x_*L*_, Flag, GFP, and β-actin antibodies. **(D)** Bcl-x_*L*_ knockdown does not affect the interaction between Mff and Drp1. Nsi or Bcl-x_*L*_ siRNA (Bcl-x_*L*_i) were transfected into HEK293 cells expressing GST or GST-Mff. GST-pulldown (GST-PD) and lysate samples were immunoblotted with Drp1, GST, Bcl-x_*L*_, and β-actin antibodies.

We have reported previously that the non−SUMOylatable Drp1 4KR mutant associates more with mitochondria than Drp1-WT (Guo et al., 2013). Consistent with those data subcellular fractionation and immunoblotting further revealed the mitochondria as a major subcellular location for the non-SUMOylatable Drp1 and Bcl-x_*L*_ interaction (Figure 4A). These results suggest an involvement of deSUMOylation-mediated Drp1 mitochondrial recruitment in regulating the Drp1-Bcl-x_*L*_ interaction. Mff is the major docking protein for Drp1 on the MOM (Otera et al., 2010) and Drp1 SUMOylation regulates mitochondrial recruitment by Mff (Guo et al., 2017). Moreover, Mff is also associated with Bcl-x_*L*_ in rat brain tissue (Li et al., 2013). We therefore investigated if the expression level of Mff is a regulatory factor for the Drp1-Bcl-x_*L*_ interaction. Mff knockdown decreased the interaction between non-SUMOylatable Drp1 and Bcl-x_*L*_ whereas Mff overexpression increased this interaction (Figures 4B,C). As expected, Bcl-x_*L*_ knockdown did not affect the binding between GST-Mff and Drp1, excluding a role for Bcl-x_*L*_ in recruiting Drp1 to the mitochondria through Mff (Figure 4D). These results suggest that Mff primes the interaction between Drp1 and Bcl-x_*L*_ most likely through recruiting non-SUMOylated Drp1 to the mitochondria, thus increasing its proximity to mitochondrial Bcl-x_*L*_.

### Mff and Bcl-x_*L*_ Directly Interact With Each Other Through Their Transmembrane Domains in a Drp1-Independent Manner

**FIGURE 5:**
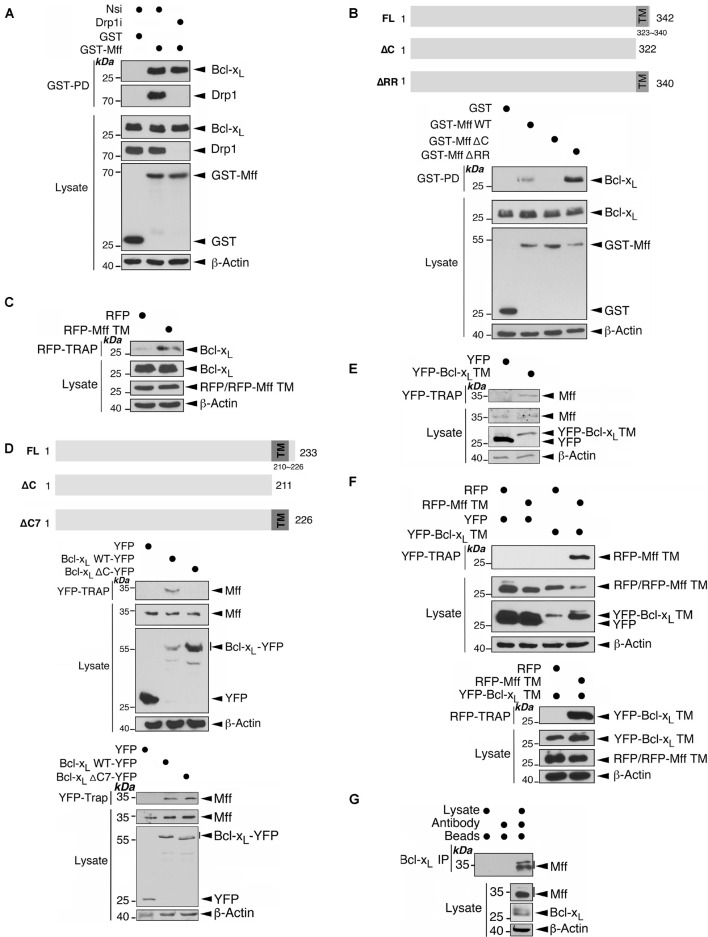
Mff and Bcl-x_*L*_ interact through their C-terminal transmembrane domains in a manner independent of Drp1. **(A)** Drp1 knockdown does not affect the interaction between Mff and Bcl-x_*L*_. Either Nsi or Drp1i were introduced into HEK293 cells expressing either GST or GST-Mff. GST-Pulldown (GST-PD) and lysate samples were immunoblotted with Bcl-x_*L*_, Drp1, GST, and β-actin antibodies. **(B)** The transmembrane domain (TM) of Mff is important for its interaction with Bcl-x_*L*_. GST, GST-Mff, GST-Mff ΔC, or GST-Mff ΔRR were transfected into HEK293 cells. GST-PD and lysate samples were immunoblotted with Bcl-x_*L*_, Drp1, GST, and β-actin antibodies. The schematics illustrate domain structures for Mff and Mff deletion mutants (Upper panel). **(C)** Mff TM interacts with Bcl-x_*L*_. RFP or RFP-Mff TM were transfected into HEK293 cells. RFP-Trap and lysate samples were immunoblotted with Bcl-x_*L*_, RFP, and β-actin antibodies. **(D)** Bcl-x_*L*_ TM is important for its interaction with Mff. The schematics illustrate domain structures for Bcl-x_*L*_, Bcl-x_*L*_ ΔC mutant and Bcl-x_*L*_ ΔC7 mutant (Upper panel). YFP, Bcl-x_*L*_-YFP, or Bcl-x_*L*_-YFP ΔC were transfected into HEK293 cells (Middle panel). YFP, Bcl-x_*L*_-YFP, or Bcl-x_*L*_-YFP ΔC7 were transfected into HEK293 cells (Lower panel). YFP-Trap and lysate samples were immunoblotted with Mff, GFP, and β-actin antibodies. **(E)** Bcl-x_*L*_ TM interacts with Mff. YFP or YFP-Bcl-x_*L*_ TM were transfected into HEK293 cells. YFP-Trap and lysate samples were immunoblotted with Bcl-x_*L*_, GFP, and β-actin antibodies. **(F)** Bcl-x_*L*_ TM and Mff TM interact. RFP or RFP-Mff TM were transfected in HEK293 expressing YFP or YFP-Bcl-x_*L*_ TM. YFP-Trap (Upper panel) or RFP-Trap (Lower panel) and lysate samples were immunoblotted with RFP, GFP, and β-actin antibodies. **(G)** Endogenous Mff is associated with endogenous Bcl-x_*L*_. Bcl-x_*L*_ was immunoprecipitated from whole cell lysates prepared from HEK293 cells using an Mff antibody. IP and lysate samples were immunoblotted with Mff, Bcl-x_*L*_, and β-actin antibodies.

An endogenous complex of Mff/Drp1/Bcl-x_*L*_ has been detected biochemically, and interactions between Mff/Drp1 and Drp1/Bcl-x_*L*_ are well established (Li et al., 2008, 2013; Otera et al., 2010; Guo et al., 2017). Therefore, we investigated if Mff can bind directly to Bcl-x_*L*_. Interestingly, Bcl-x_*L*_ was associated with GST-Mff in HEK293 cells, and this association was not affected by Drp1 depletion by RNAi-mediated knockdown, suggesting Mff and Bcl-x_*L*_ may interact independently of Drp1 (Figure 5A). Next, we examined the interfaces involved in Mff-Bcl-x_*L*_ interactions. The first 50 amino acid residues at the N-terminus of Mff, which mediate recruitment of Drp1 to the mitochondria (Otera et al., 2010), are not important for binding of GST-Mff to Bcl-x_*L*_ (Supplementary Figure 2). In contrast, removal of the last 20 amino acids of Mff, containing the transmembrane domain (TM of 18 residues), plus 2 C-terminal residues located in the mitochondrial intermembrane space (IMS), abolished the binding of the two proteins. Deletion of only the 2 IMS residues increased the binding of GST-Mff to Bcl-x_*L*_ (Figure 5B), suggesting that the Mff TM is essential for the Mff-Bcl-x_*L*_ interaction and that the presence of two IMS-located residues may inhibit this interaction through an unknown mechanism. Indeed, RFP-Mff TM interacts with Bcl-x_*L*_ (Figure 5C), confirming the importance of the Mff TM in mediating the Mff-Bcl-x_*L*_ interaction. Likewise, removal of the last 22 amino acid residues of Bcl-x_*L*_, containing most of the TM domain (15 residues of TM) plus 7 residues present in the IMS, abolished binding between the two proteins. Deletion of the 7 IMS residues did not alter the binding of Bcl-x_*L*_-YFP to Mff (Figure 5D), suggesting that the TM of Bcl-x_*L*_ is also important for the Mff-Bcl-x_*L*_ interaction. Indeed, YFP-Bcl-x_*L*_ TM interacts with Mff (Figure 5E), confirming the importance of the Bcl-x_*L*_ TM in mediating the Mff-Bcl-x_*L*_ interaction. Furthermore, the TMs of both proteins interacted with each other (Figure 5F). Finally, we detected endogenous association between Mff and Bcl-x_*L*_ in untreated HEK293 cells (Figure 5G). These results demonstrate a TM-mediated interaction between Mff and Bcl-x_*L*_ which occurs independently of Drp1.

### Effects of Cell Stress on Drp1 Recruitment in HEK293 Cells

**FIGURE 6:**
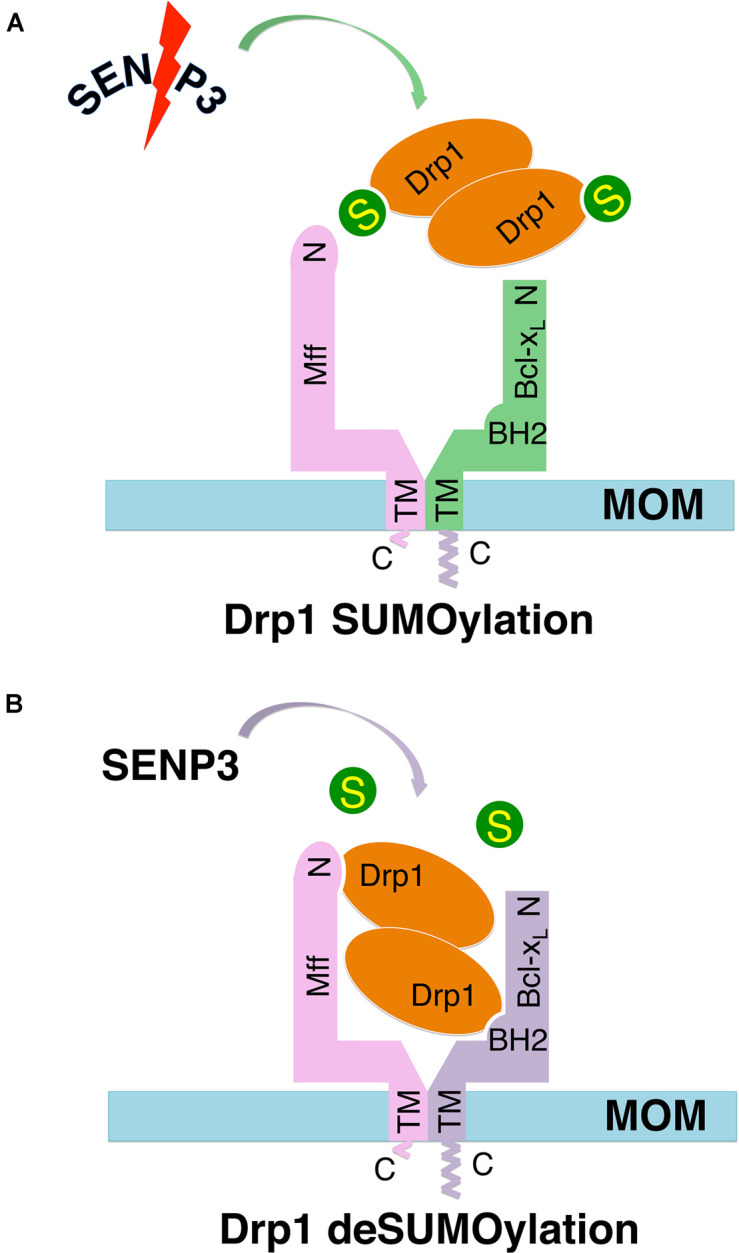
Schematics of hypothetical models show the Mff-primed Bcl-x_*L*_-Drp1 interaction regulated by the SUMOylation status of Drp1. In these hypothetical models, Mff recruits self-associated (dimeric and/or tetrameric) Drp1 to the mitochondrial outer membrane (MOM), bringing Drp1 in close proximity to Bcl-x_*L*_, which also interacts with the Mff TM through its C-terminus, thereby priming the Bcl-x_*L*_-Drp1 interaction. **(A)** SUMOylation decreases Drp1 availability on the MOM via reducing Mff-mediated recruitment, and also reduces the direct interaction between Bcl-x_*L*_ and Drp1. **(B)** deSUMOylation increases Drp1 availability on the MOM via promoting Mff-mediated recruitment, and also enhances the direct interaction between Bcl-x_*L*_ and Drp1.

We have previously reported that in cultured primary neurons metabolic stress caused by oxygen-glucose deprivation (OGD) cause a reduction in SENP3 levels that enhances Drp1 SUMOylation by SUMO-2/3, leading to Drp1 partitioning away from mitochondria (Guo et al., 2013). However, OGD plus reoxygenation promoted SENP3 recovery, Drp1 deSUMO-2/3-ylation, Drp1 recruitment to mitochondria and consequent loss of mitochondrial integrity (Guo et al., 2013). Consistent with those results, here we show that OGD plus reoxygenation reduced Drp1 deSUMO-2/3-ylation beneath control levels in HEK293 cells (Supplementary Figure 3). Taken together, our previous finding that SENP3-mediated deSUMOylation enhances Drp1 mitochondrial recruitment through selectively promoting the Drp1-Mff interaction (Guo et al., 2017) and that a region in BH2 domain of Bcl-x_*L*_ is essential for its interaction with Drp1 (Li et al., 2013), we propose a model (Figure 6) whereby an Mff-primed Drp1-Bcl-x_*L*_ interaction is regulated by the SUMOylation status of Drp1.

### Oxygen-Glucose Deprivation Reduces the Drp1-Bcl-x_*L*_ Interaction While Oxygen-Glucose Deprivation Plus Reoxygenation Enhances It

**FIGURE 7:**
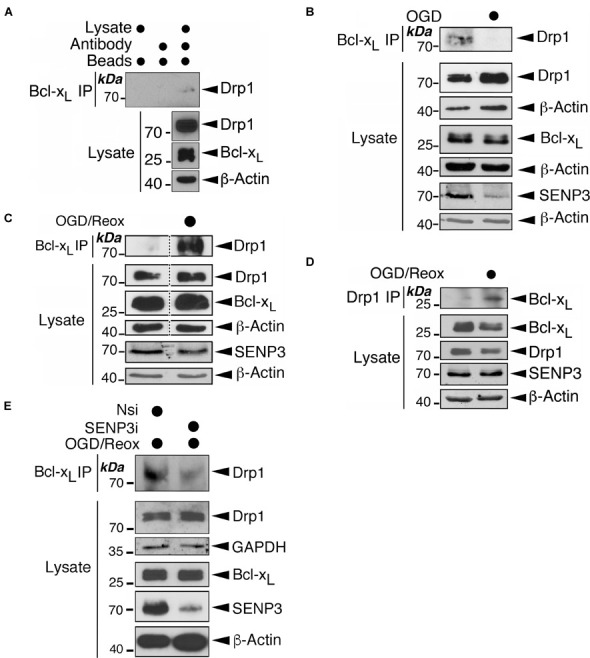
Exposure of cells to OGD or OGD plus reoxygenation results in changes in the Drp1-Bcl-x_*L*_ association. **(A)** Endogenous Bcl-x_*L*_ is associated with endogenous Drp1. Bcl-x_*L*_ was immunoprecipitated from whole cell lysates prepared from HEK293 cells using a Bcl-x_*L*_ antibody. IP and lysate samples were immunoblotted with Drp1, Bcl-x_*L*_, and β-actin antibodies. **(B)** Exposure of cells to OGD results in decreased association between Bcl-x_*L*_ and Drp1. HEK293 cells were subjected to OGD for 2 h. At the end of OGD the cells were harvested and lysates were immunoprecipitated for Bcl-x_*L*_. **(C)** Exposure of cells to OGD plus reoxygenation results in increased association between Bcl-x_*L*_ and Drp1. HEK293 cells were subjected to OGD for 2 h followed by 24 h reoxygenation. At the end of reoxygenation the cells were harvested and lysates were immunoprecipitated for Bcl-x_*L*_. Removal of unrelated lanes indicated by dotted lines. **(D)** Exposure of cells to OGD plus reoxygenation results in increased association between Bcl-x_*L*_ and Drp1. HEK293 cells were subjected to OGD for 2 h followed by 24 h reoxygenation. At the end of reoxygenation the cells were harvested and lysates were immunoprecipitated for Drp1. **(E)** SENP3 knockdown reduces the association between Bcl-x_*L*_ and Drp1 induced by OGD plus reoxygenation. HEK293 cells were transfected with Nsi or SENP3i for 48 h before being subjected to OGD for 2 h followed by 24 h reoxygenation. At the end of reoxygenation the cells were harvested and lysates were immunoprecipitated for Bcl-x_*L*_. In (B–E) IP and lysate samples were immunoblotted with Drp1, Bcl-x_*L*_, SENP3, GAPDH and β-actin antibodies.

To interrogate this proposal further, we investigated if OGD-induced changes on SENP3 levels impact on the endogenous association of Bcl-x_*L*_-Drp1. Indeed, while Drp1 associated with Bcl-x_*L*_ in unstressed cells (Figure 7A and Supplementary Figure 4), this association was greatly reduced in cells subjected to OGD alone (Figure 7B). In contrast, the association between the two proteins was markedly enhanced in reoxygenated cells after OGD (Figures 7C,D). Importantly, SENP3 knockdown in reoxygenated cells after OGD reduced the Bcl-x_*L*_-Drp1 interaction (Figure 7E). Collectively these results suggest that alterations in SENP3 levels, and consequent changes to the SUMOylation status of Drp1, regulate the Bcl-x_*L*_-Drp1 association following ischemic insult.

### The Drp1-Bcl-x_*L*_ Interaction Promotes Cell Death Following Reoxygenation

**FIGURE 8:**
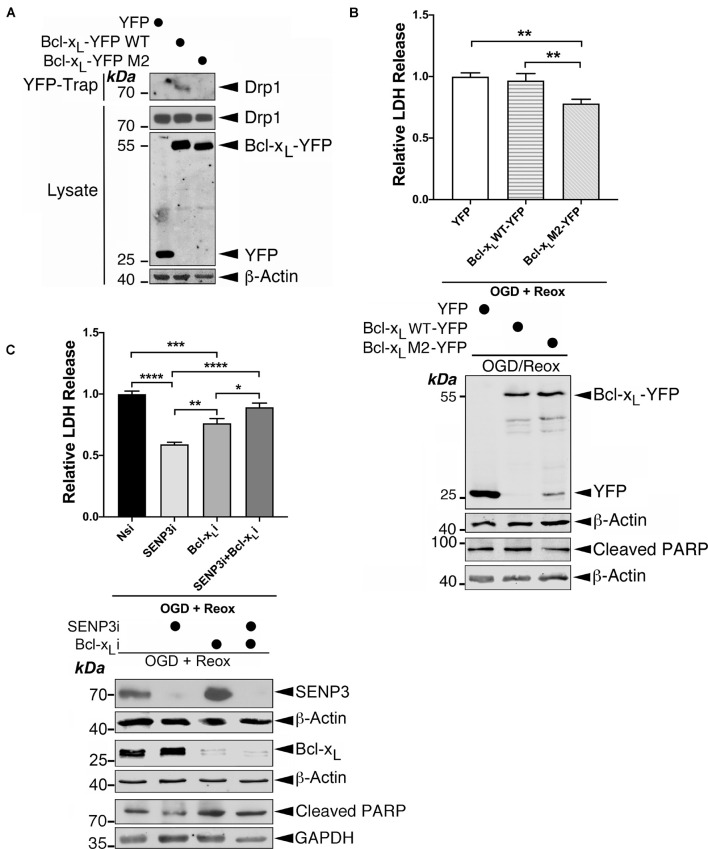
The Drp1-Bcl-x_*L*_ interaction contributes to cell death following reoxygenation. **(A)** The Bcl-x_*L*_ M2 mutant does not interact with endogenous Drp1. YFP, Bcl-x_*L*_-YFP or Bcl-x_*L*_ M2 (mutant)-YFP were transfected into HEK293 cells. YFP-Trap and lysate samples were immunoblotted with Drp1, GFP, and β-actin antibodies. **(B)** Expression of Bcl-x_*L*_ M2-YFP decreases OGD plus reoxygenation-evoked LDH release. Immunoblots (lower panel) show expression of YFP, Bcl-x_*L*_-YFP or Bcl-x_*L*_-YFP M2 mutant in HEK293 cells subjected to OGD plus reoxygenation and cleaved PARP in these cells. **(C)** Bcl-x_*L*_ knockdown reduces OGD plus reoxygenation-evoked LDH release in the presence of SENP3 but not in the absence of SENP3. Immunoblots (lower panel) show knockdown of SENP3 and/or Bcl-x_*L*_ in HEK293 cells subjected to OGD plus reoxygenation and cleaved PARP in these cells. In **(B,C)** values are presented as mean ± SEM in the histogram (*n* = 4∼6 replicates for each group; *p < 0.05; **p < 0.01; ***p < 0.001; ****p < 0.0001).

To examine the role for the Drp1-Bcl-x_*L*_ interaction in OGD plus reoxygenation-evoked LDH release, a marker for cell death, we used a Bcl-x_*L*_ W188S/D189V/F191C mutant (Li et al., 2013) that does not bind Drp1 (Figure 8A). Expressing this Drp1 binding-defective Bcl-x_*L*_ mutant, but not the WT counterpart, significantly reduced OGD plus reoxygenation-evoked LDH release by ∼22% (Figure 8B), indicating a contributory role for the Drp1-Bcl-x_*L*_ interaction in cell death following ischemia. We have previously shown that SENP3 and Drp1 act in the same pathway to promote cell death following ischemia. Knockdown of either protein reduces OGD plus reoxygenation-evoked LDH release, and knockdown of both proteins has no additive protective effect (Guo et al., 2013). Therefore, we investigated the effects of knocking down either SENP3 or Bcl-x_*L*_, or both in cells later subjected to reoxygenation following OGD. Consistent with our previous findings (Guo et al., 2013, 2017), SENP3 knockdown is cytoprotective for cells subjected to OGD plus reoxygenation as evidenced by significantly decreased LDH levels and decreased cleaved PARP levels (a marker for caspase-dependent apoptosis) (Figure 8C). Bcl-x_*L*_ knockdown also significantly reduced OGD plus reoxygenation-evoked LDH release by ∼24%, indicating a pro-death role for this Bcl-2 family member in this *in vitro* model of ischemia (Figure 8C).

Interestingly, knockdown of either SENP3 or Bcl-x_*L*_ abolished the protective effects observed upon the knockdown of either protein (Figure 8C), suggesting that SENP3 and Bcl-x_*L*_ both have required roles in cell death following reoxygenation. Bcl-x_*L*_ may act in other pathway(s) in conjunction with, or in parallel with, the pathway controlled by SENP3-mediated deSUMOylation of Drp1. Collectively, these results indicate that the Drp1-Bcl-x_*L*_ interaction promotes cell death following reoxygenation after OGD and suggest that Bcl-x_*L*_ has additional role(s) in regulating cell death through unknown mechanism(s).

## Discussion

There is a wealth of evidence that SUMOlyation can play a central role in cytoprotection (for review see Guo and Henley, 2014), but the specific components in these adaptive cell responses remain largely unidentified. In this study, we report that Drp1 SUMOylation regulates the interaction between Drp1 and Bcl-x_*L*_ that contributes to the loss of mitochondrial viability and cell death evoked by reoxygenation following OGD.

Mff is the major docking protein for Drp1 recruitment to the MOM. Our data demonstrate endogenous association between the transmembrane domains of Mff and Bcl-x_*L*_. Our results are consistent with a stoichiometric ratio of 1:1 at mitochondria for exogenously expressed Mff and Bcl-x_*L*_ reported previously using fluorescence resonance energy transfer two-hybrid analysis (Ma et al., 2019). Moreover, our findings suggest the Mff-Bcl-x_*L*_ interaction may serve as a scaffolding complex for facilitating the Drp1-Bcl-x_*L*_ interaction.

We find that SENP3 promotes the Drp1-Bcl-x_*L*_ interaction through at least two mechanisms: (i) an indirect pathway where SENP3-mediated deSUMOylation leads to increased mitochondrial recruitment of Drp1 through enhanced Drp1-Mff interaction, and (ii) a direct pathway where SENP3-mediated deSUMOylation facilitates the Drp1-Bcl-x_*L*_ interaction.

Furthermore, in addition to Bcl-x_*L*_ localized in the mitochondria through its TM domain, the Bcl-2 protein is also present in the cytosol (Edlich et al., 2011). Because the binding site for Bcl-x_*L*_ to Drp1 is outside the TM domain, the cytosolic Bcl-x_*L*_ may also interact with Drp1 and this may also be facilitated by SENP3-mediated deSUMOylation. For example, a Drp1-Bcl-x_*L*_ interaction has been detected in the synaptic vesicles of cultured rat hippocampal neurons where non-mitochondrial Mff is responsible for recruiting Drp1 to synaptic vesicles through an unidentified mechanism (Li et al., 2008, 2013). Therefore, it is tempting to speculate that SENP3 might have a role in the regulation of Drp1-Bcl-x_*L*_-mediated synaptic vesicle membrane dynamics during endocytosis in neurons.

We note that in the present study that although SENP3 recovered to control levels in cells subjected to reoxygenation after OGD the Drp1-Bcl-x_*L*_ interaction remained enhanced. Based on our experimental evidence presented in this study showing that (i) SENP3 levels return to approximately control levels (Figures 7C,D), and (ii) Drp1 SUMO-2/3-ylation levels actually decrease beneath control levels (Supplementary Figure 3), in the reoxygenationed cells after OGD, there are two scenarios to be further explored in our future investigation: (i) recovered SENP3 upon reoxygenation is more active in deSUMO-2/3-ylating Drp1, and (ii) other factors resulting in decreased SUMO-2/3-conjugation upon reoxygenation contribute to this reduction of Drp1 SUMO-2/3-ylation. Thus, a tenable explanation for this observation might be that the recovery of SENP3, in addition to possible other factors, leads to a “rebound” in Drp1 SUMOylation beneath control levels, leading to enhanced Drp1-Bcl-x_*L*_ interaction.

Interestingly, we find that RNAi-mediated depletion of Bcl-x_*L*_ reduces cell death evoked by OGD plus reoxygenation. This indicates that Bcl-x_*L*_ plays an overall pro-cell death role in our OGD/reoxygenation model. Two possibilities have been considered. First, Bcl-x_*L*_ is normally anti-apoptotic but cleavage or occlusion of the N-terminus could transform it into a pro-cell death fragment that acts like pro-cell death protein Bax (Jonas et al., 2004). However, if this were the case in our OGD/reoxygenation model, a Bcl-x_*L*_ cleavage fragment of ∼6∼8 kDa would be expected. We were unable to detect any such cleavage product using an N-terminal directed rabbit monoclonal antibody for Bcl-x_*L*_ (54H6; Cell Signaling) (Supplementary Figure 5). Moreover, expressing YFP-tagged Bcl-x_*L*_ lacking amino acids 2–76 did not cause changes in either cleaved PARP levels or LDH release levels in HEK293 cells subjected to OGD plus reoxygenation (Supplementary Figure 6). Nevertheless, in future studies it may be necessary to perform other methods, such as mass spectrometry, to further address the question of whether Bcl-x cleavage occurs in this model system.

A second possibility is that the Drp1-Bcl-x_*L*_ interaction or additional protein(s) binding to the complex may cause conformational changes in Bcl-x_*L*_ and turn it into a pro-cell death factor. It has been reported previously that conformational changes of Bcl-x_*L*_ can be induced by its association with Bcl-2 homology 3 (BH3)-only Protein p53 Up-regulated Modulator of Apoptosis (PUMA) (Follis et al., 2013), which appears to mediate mitochondrial localization of Drp1 upon I/R injury (Din et al., 2013). It would be very interesting to investigate in future if PUMA involvement underlies the Bcl-x_*L*_ pro-cell death effect revealed in this study.

Intriguingly, we found that Bcl-x_*L*_ depletion has a similar effect to expressing a Drp1-binding defective Bcl-x_*L*_ mutant in cells subjected to OGD plus reoxygenation, suggesting that loss of the Drp1-Bcl-x_*L*_ interaction following Bcl-x_*L*_ depletion might result in this cytoprotective effect. However, it should be noted that Bcl-x_*L*_ plays fundamental roles in controlling multiple cellular functions including regulation of cell death and survival, autophagy, maintenance of homeostatic metabolism, and coordination of adaptive responses to stress, via innumerable mechanisms (Michels et al., 2013). Loss of Bcl-x_*L*_ would result in cellular landscape changes in many respects including the basal levels of mitochondrial fission and fusion, bioenergetic capacity and membrane integrity. Therefore, in this respect, the loss of the Drp1-Bcl-x_*L*_ interaction would be one of a myriad of landscape changes upon RNAi-mediated depletion of Bcl-x_*L*_, and might not be the reason underlying the cytoprotective effect. Moreover, RNAi-mediated depletion of Bcl-x_*L*_ does not affect the Drp1-Mff interaction that is substantially responsible for Drp1 recruitment (Otera et al., 2010; Loson et al., 2013) and is regulated by SENP3-mediated deSUMOylation (Guo et al., 2017). However, a puzzle remains as to the reason why in the absence of Bcl-x_*L*_, RNAi-mediated SENP3 depletion does not appear to prevent cell death evoked by reoxygenation following OGD. One plausible explanation could be that changes caused by Bcl-x_*L*_ loss may offset the cytoprotective effect resulting from reduced Mff-mediated Drp1 mitochondrial localization due to SENP3 knockdown in cells subjected to OGD plus reoxygenation. In other words, in the OGD/reoxygenation model used in this study loss of Bcl-x_*L*_ is, on one hand a benefit for cell survival but, on the other hand might greatly affect unknown component(s)/parameters/pathway(s) essential for the protective effect of SENP3-mediated deSUMOylation.

## Data Availability Statement

The original contributions presented in the study are included in the article/Supplementary Material, further inquiries can be directed to the corresponding authors.

## Author Contributions

CG conceived the project, designed, and performed most biochemical and molecular biological experiments. KH performed cell-imaging experiments. JJ conducted mutagenesis work. AZ contributed to cell culture and transfection work. CG and KW provided team leadership and project management. CG wrote the manuscript with the contribution from WG, JH, and KW in review and editing. All authors contributed to hypothesis development, experimental design and data interpretation.

## Conflict of Interest

The authors declare that the research was conducted in the absence of any commercial or financial relationships that could be construed as a potential conflict of interest.

## Publisher’s Note

All claims expressed in this article are solely those of the authors and do not necessarily represent those of their affiliated organizations, or those of the publisher, the editors and the reviewers. Any product that may be evaluated in this article, or claim that may be made by its manufacturer, is not guaranteed or endorsed by the publisher.
