# Supplementary material for: SENP3 Promotes an Mff-Primed Bcl-xL-Drp1 Interaction Involved in Cell Death Following Ischemia
Source: Front Cell Dev Biol. 2021 Oct 15;9:752260. doi: 10.3389/fcell.2021.752260 (PMC8555761; doi:10.3389/fcell.2021.752260)
Supplement: Supplementary file 1 [file Data_Sheet_1.pdf]

frontiers

Supplementary Figures 1-6

SENP3 promotes an Mff-primed Bcl-x_L-Drp1 interaction involved in cell death following ischemia

Chun Guo[1]*, Keri L Hildick[2], Juwei Jiang[1], Alice Zhao[1], Wenbin Guo[3], Jeremy M Henley[2,4] and Kevin A Wilkinson[2]*

[1]School of Biosciences, University of Sheffield, Sheffield, United Kingdom

[2]School of Biochemistry, University of Bristol, Bristol, United Kingdom

[3]School of Medical Sciences, University of Manchester, Manchester, United Kingdom

[4]Centre for Neuroscience and Regenerative Medicine, Faculty of Science, University of Technology Sydney, Ultimo, NSW, Australia

* These authors share senior authorship

*** Correspondence:**
Corresponding Author Chun Guo or Kevin A Wilkinson
c.guo@sheffield.ac.uk or Kevin.Wilkinson@bristol.ac.uk

Keywords: Drp1, Bcl-x_L, SENP3, Mff, SUMOylation, ischemia

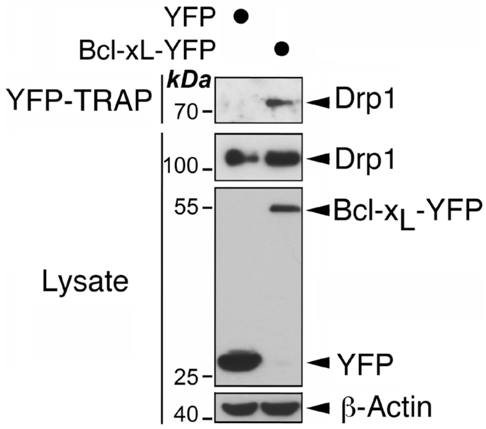

Supplementary Figure 1. Endogenous Drp1 is associated with cell-expressed Bcl-x_L. Either YFP or Bcl-x_L-YFP was introduced into HEK293 cells. Lysates were subjected to pulldown using YFP-Trap for YFP or YFP-tagged Bcl-x_L. YFP-Trap and lysate samples were immunoblotted with Drp1, GFP, and β-actin antibodies.

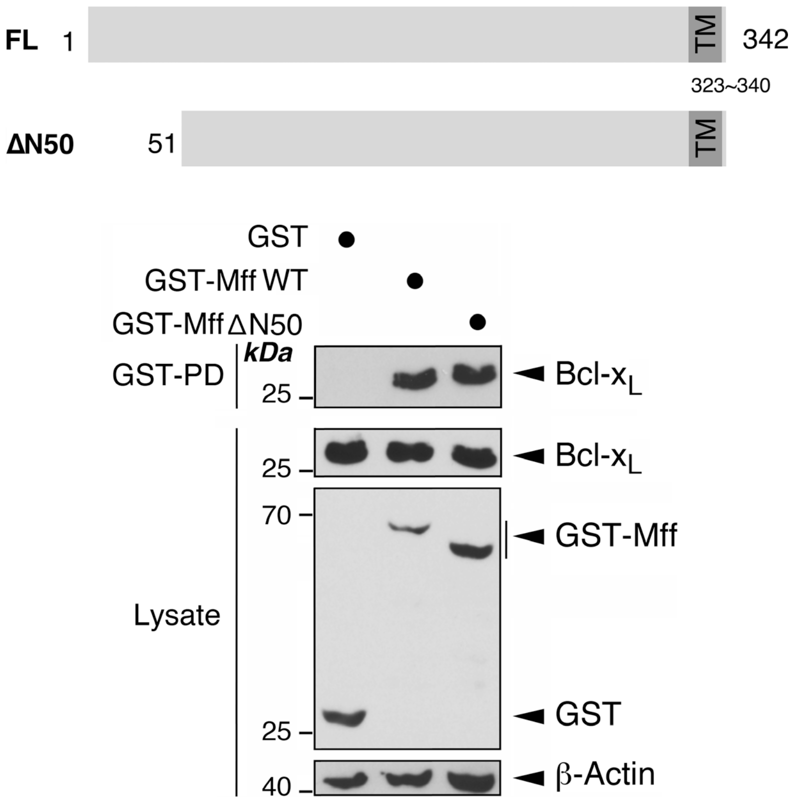

Supplementary Figure 2. 1-50aa in the N-terminus of Mff is not required for the Mff-Bcl-x_L interaction. GST, GST-Mff, or GST-Mff ΔN50 were transfected into HEK293 cells. GST-PD and lysate samples were immunoblotted with Bcl-x_L, GST, and β-actin antibodies. The schematics illustrate domain structure for Mff and Mff ΔN50 mutant (Upper panel).

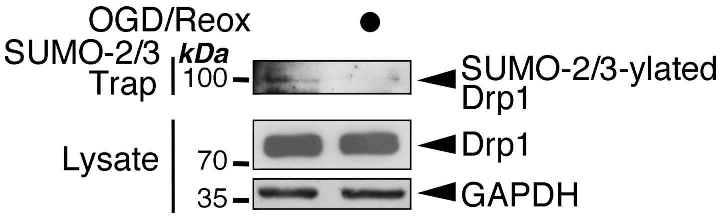

Supplementary Figure 3. OGD plus reoxygenation results in Drp1 deSUMO-2/3-ylation. HEK293 cells were subjected to OGD for 2 h followed by 24 h reoxygenation. At the end of reoxygenation the cells were harvested and SUMO-2/3 conjugates were purified using SUMO-2/3-Trap beads. SUMO-2/3-Trap and lysate samples were immunoblotted with Drp1 and GAPDH antibodies.

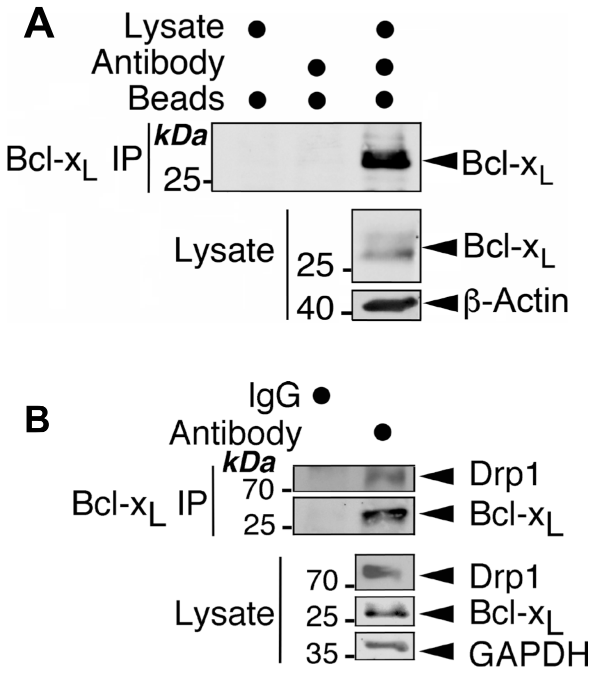

Supplementary Figure 4. Drp1 is associated with Bcl-x_L in HEK293 cells. *A*) Immunoprecipitation of Bcl-x_L was carried out using a rabbit monoclonal antibody bound to protein A beads from HEK293 cells. *B*) Drp1 is associated with precipitated Bcl-x_L from HEK293 cells. IP and lysate samples were immunoblotted with Bcl-x_L, Drp1, β-actin and GAPDH antibodies.

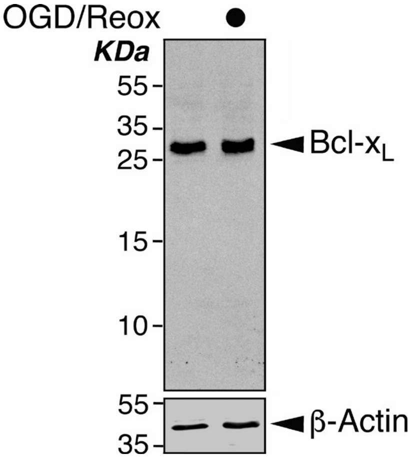

Supplementary Figure 5. Reoxygenation following OGD does not seem to result in apparent cleavage of Bcl-x_L. HEK293 cells subjected to OGD plus reoxygenation. Lysate samples were separated on a 15% resolving gel and immunoblotted with Bcl-x_L and β-actin antibodies.

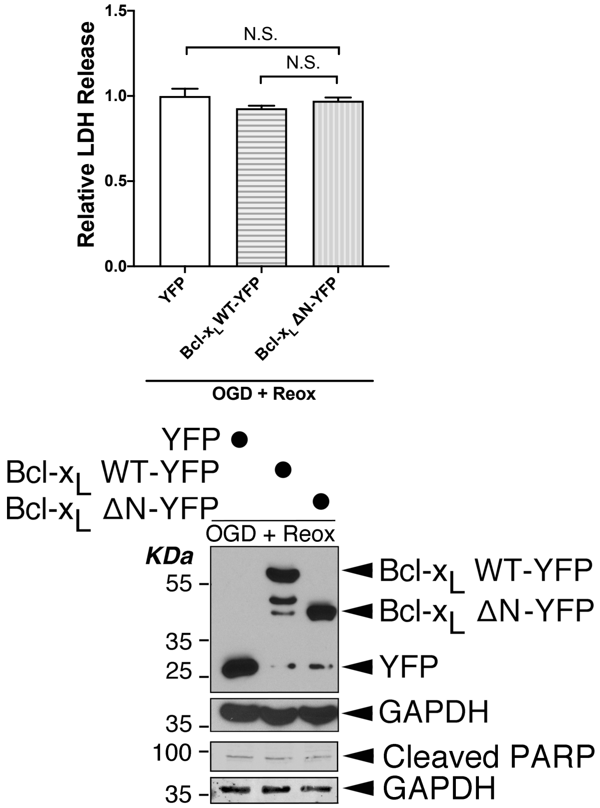

Supplementary Figure 6. Expressing YFP-Bcl-x_L lacking amino acids 2-76 does not cause significant changes in LDH release in HEK293 cells subjected to reoxygenation following OGD. Immunoblots (lower panel) shows the levels of cleaved PARP in HEK293 cells expressing YFP, Bcl-x_L-YFP or Bcl-x_L ΔN-YFP mutant following OGD plus reoxygenation. Values are presented as mean ± SEM in the histogram (n=6 replicates for each group; N.S., not significant, $p>0.05$).
